# Insulin resistance and metabolic health predict cardiorespiratory fitness: cohort study

**DOI:** 10.1093/ehjopen/oeag029

**Published:** 2026-02-25

**Authors:** Liliana Muñoz-Hernandez, Ana Leonor Rivera, Adrian Soto-Mota, Jesus Paez-Mayorga, Jesus Flores-Brito, Erick Resendiz-Carrillo, Guillermo Roa-Alvarez, Leticia Lopez-Carreola, Sebastian Zamora-Gutierrez, Perla Alpizar-Chacon, Eunice Barbosa-Meillon, Antonio Barajas-Martínez, Ivette Cruz-Bautista, Gabriela A Galan-Ramírez, Donaji Veronica Gomez-Velasco, Fabiola Mabel del Razo-Olvera, Carlos A Aguilar-Salinas

**Affiliations:** Unidad de Investigación de Enfermedades Metabólicas, Instituto Nacional de Ciencias Médicas y Nutrición Salvador Zubirán, Vasco de Quiroga 15, Tlalpan, Mexico City 14080, Mexico; Secretaría de Ciencia, Humanidades, Tecnología e Innovación (SECIHTI), Insurgentes Sur 1582 , Benito Juarez, Mexico City 03940, Mexico; Department of Endocrinology and Metabolism, Instituto Nacional de Ciencias Médicas y Nutrición Salvador Zubirán, Vasco de Quiroga 15, Tlalpan, Mexico City 14080, Mexico; School of Medicine and Health Sciences, Tecnologico de Monterrey, Calle del puente 222, Tlalpan, Mexico City 14830, Mexico; Unidad de Investigación de Enfermedades Metabólicas, Instituto Nacional de Ciencias Médicas y Nutrición Salvador Zubirán, Vasco de Quiroga 15, Tlalpan, Mexico City 14080, Mexico; Instituto de Ciencias Nucleares, Universidad Nacional Autónoma de México, Circuito exterior SN, Coyoacan, Mexico City 04510, Mexico; Unidad de Investigación de Enfermedades Metabólicas, Instituto Nacional de Ciencias Médicas y Nutrición Salvador Zubirán, Vasco de Quiroga 15, Tlalpan, Mexico City 14080, Mexico; School of Medicine and Health Sciences, Tecnologico de Monterrey, Calle del puente 222, Tlalpan, Mexico City 14830, Mexico; Unidad de Investigación de Enfermedades Metabólicas, Instituto Nacional de Ciencias Médicas y Nutrición Salvador Zubirán, Vasco de Quiroga 15, Tlalpan, Mexico City 14080, Mexico; School of Medicine and Health Sciences, Tecnologico de Monterrey, Calle del puente 222, Tlalpan, Mexico City 14830, Mexico; Scholarship Holder of the Dirección General de Calidad y Educación en Salud, Secretaria de Salud, Marina Nacional 60, Miguel Hidalgo, Mexico City 11400, Mexico; Unidad de Investigación de Enfermedades Metabólicas, Instituto Nacional de Ciencias Médicas y Nutrición Salvador Zubirán, Vasco de Quiroga 15, Tlalpan, Mexico City 14080, Mexico; Unidad de Investigación de Enfermedades Metabólicas, Instituto Nacional de Ciencias Médicas y Nutrición Salvador Zubirán, Vasco de Quiroga 15, Tlalpan, Mexico City 14080, Mexico; School of Medicine and Health Sciences, Tecnologico de Monterrey, Calle del puente 222, Tlalpan, Mexico City 14830, Mexico; Unidad de Investigación de Enfermedades Metabólicas, Instituto Nacional de Ciencias Médicas y Nutrición Salvador Zubirán, Vasco de Quiroga 15, Tlalpan, Mexico City 14080, Mexico; School of Medicine and Health Sciences, Tecnologico de Monterrey, Calle del puente 222, Tlalpan, Mexico City 14830, Mexico; Unidad de Investigación de Enfermedades Metabólicas, Instituto Nacional de Ciencias Médicas y Nutrición Salvador Zubirán, Vasco de Quiroga 15, Tlalpan, Mexico City 14080, Mexico; Unidad de Investigación de Enfermedades Metabólicas, Instituto Nacional de Ciencias Médicas y Nutrición Salvador Zubirán, Vasco de Quiroga 15, Tlalpan, Mexico City 14080, Mexico; School of Medicine and Health Sciences, Tecnologico de Monterrey, Calle del puente 222, Tlalpan, Mexico City 14830, Mexico; School of Medicine and Health Sciences, Tecnologico de Monterrey, Calle del puente 222, Tlalpan, Mexico City 14830, Mexico; School of Medicine and Health Sciences, Tecnologico de Monterrey, Calle del puente 222, Tlalpan, Mexico City 14830, Mexico; Unidad de Investigación de Enfermedades Metabólicas, Instituto Nacional de Ciencias Médicas y Nutrición Salvador Zubirán, Vasco de Quiroga 15, Tlalpan, Mexico City 14080, Mexico; School of Medicine and Health Sciences, Tecnologico de Monterrey, Calle del puente 222, Tlalpan, Mexico City 14830, Mexico; Centro de Ciencias de la Complejidad (C3), Universidad Nacional Autónoma de México, Circuito Mario de la Cueva 20, Coyoacan, Mexico City 04510, Mexico; Estancias Posdoctorales por México, CONAHCYT, Insurgentes Sur 1582 , Benito Juarez, Mexico City 03940, Mexico; Unidad de Investigación de Enfermedades Metabólicas, Instituto Nacional de Ciencias Médicas y Nutrición Salvador Zubirán, Vasco de Quiroga 15, Tlalpan, Mexico City 14080, Mexico; Department of Endocrinology and Metabolism, Instituto Nacional de Ciencias Médicas y Nutrición Salvador Zubirán, Vasco de Quiroga 15, Tlalpan, Mexico City 14080, Mexico; Unidad de Investigación de Enfermedades Metabólicas, Instituto Nacional de Ciencias Médicas y Nutrición Salvador Zubirán, Vasco de Quiroga 15, Tlalpan, Mexico City 14080, Mexico; Unidad de Investigación de Enfermedades Metabólicas, Instituto Nacional de Ciencias Médicas y Nutrición Salvador Zubirán, Vasco de Quiroga 15, Tlalpan, Mexico City 14080, Mexico; Unidad de Investigación de Enfermedades Metabólicas, Instituto Nacional de Ciencias Médicas y Nutrición Salvador Zubirán, Vasco de Quiroga 15, Tlalpan, Mexico City 14080, Mexico; Unidad de Investigación de Enfermedades Metabólicas, Instituto Nacional de Ciencias Médicas y Nutrición Salvador Zubirán, Vasco de Quiroga 15, Tlalpan, Mexico City 14080, Mexico; School of Medicine and Health Sciences, Tecnologico de Monterrey, Calle del puente 222, Tlalpan, Mexico City 14830, Mexico

**Keywords:** Cardiopulmonary exercise test, Cardiorespiratory fitness, VO_2max_, HOMA-IR, Insulin resistance, QUICKI

## Abstract

**Aims/aims:**

Decreased cardiorespiratory fitness (CRF) is an all-cause mortality predictor. Oxygen consumption at peak exercise (VO_2max_) during a cardiopulmonary exercise test (CPET) is the gold standard for its evaluation. Since cardiometabolic risk factors reduce CRF, we aimed to assess the cardiopulmonary and metabolic responses during CPET and evaluate their determinants.

**Methods and results:**

Subjects underwent incremental treadmill CPET and bioelectrical impedance analysis. Insulin sensitivity was estimated using the HOMA, QUICKI, and METS-IR indices. Multivariate regressions were used to evaluate determinants of VO_2max_. Nonlinearity was confirmed with an F-test between linear and polynomial models. Five hundred and three subjects were evaluated, 474 met maximum effort criteria, (64% females). Median age was 4(26–52); 41% had normal weight, 33% overweight, 26% obesity. Prevalence of insulin resistance ranged from 22% to 46%, depending on the equation used. VO_2max_ was 29.8(24–36) and 36.6 (30.6–43.3) mL/kg/min for females and males. Body composition analysis revealed that a higher BMI exhibited strong biological collinearity with metrics associated with adiposity excess and was inversely associated with CRF. After adjusting for age, sex, BMI, and fat mass, insulin resistance evaluated by QUICKI explained up to 43% of VO_2max_ variability and was inversely associated with CRF.

**Conclusion:**

In a cohort of individuals without established CVD, the main determinants of CRF were modifiable risk factors associated with excess adiposity and insulin resistance. The potential mechanisms underlying the reduction in CRF include decreased relative muscle mass and insulin resistance, which reduce muscle glucose uptake and O_2_ consumption during maximum effort, where anaerobic glycolysis plays a central role.

## Structured graphical abstract

**Structured graphical abstract:**
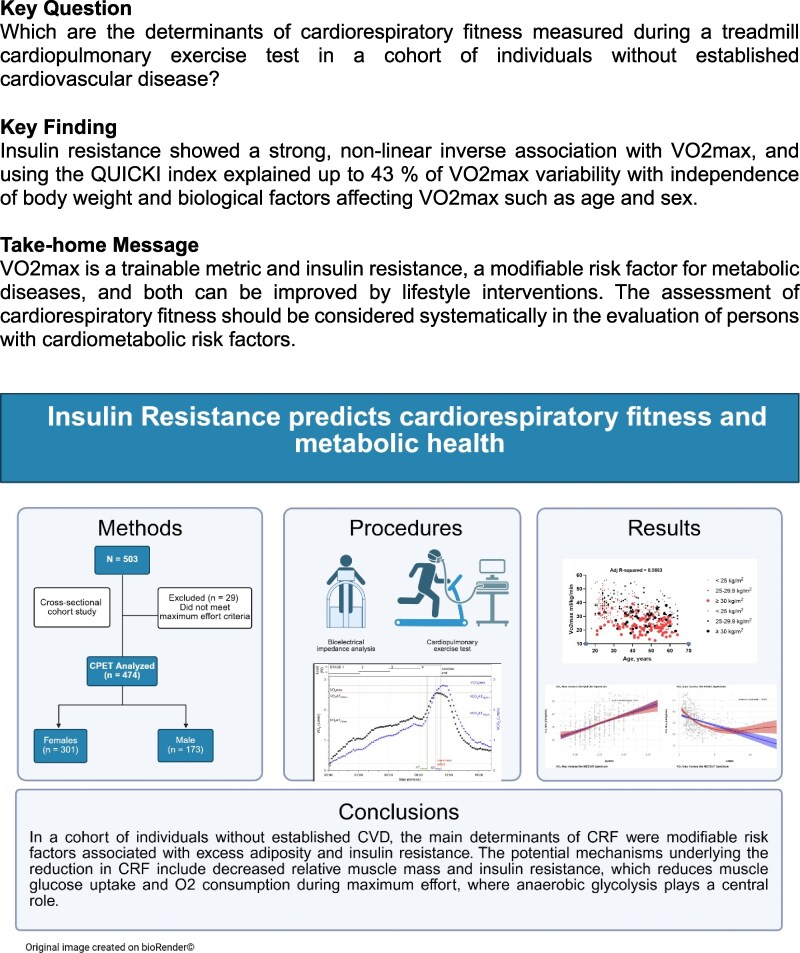

## Introduction

Maximum oxygen consumption (VO_2max_) is inversely associated with cardiovascular risk (CVR) and is an independent predictor of all-cause mortality.^1–3^ VO_2max_, a measure of cardiorespiratory fitness (CRF), refers to the integrated ability to uptake and deliver oxygen to body tissues, mainly skeletal muscle, during exercise, and the gold standard for its assessment is the cardiopulmonary exercise test (CPET) with incremental exercise protocols^4,5^

The substantial clinical implications of a decreased CRF have even prompted the proposal of VO_2max_ as a new vital sign.^6^ CRF is reduced in conditions affecting the cardiovascular and pulmonary systems, and these reductions have been widely described in individuals with heart failure and restrictive and obstructive lung diseases^7–9^. Notably, CRF is decreased in individuals with cardiometabolic risk factors such as obesity, diabetes, and low HDL-cholesterol (HDL-C), which is congruent with the associated risk for CVD.^10–15^ One underlying mechanism linking these cardiometabolic risk factors is insulin resistance.^12,13,16–19^

On the other hand, the euglycemic–hyperinsulinaemic clamp is the gold standard for insulin sensitivity assessment.^20^ However, due to the complexity of this method, multiple equation-based methods have been developed. The homeostatic model assessment for insulin resistance (HOMA-IR) and quantitative insulin sensitivity check index (QUICKI) are validated methods for assessing insulin resistance; however, they rely on direct insulin measurements, which are expensive and highly variable depending on the immunoassay technique used^21,22^ The metabolic score for insulin resistance (METS-IR) is an indirect method for detecting insulin resistance, validated against the euglycemic-hyperinsulinaemic clamp in the Mexican population.^23^ METS-IR does not require fasting insulin measurement. Instead, it utilizes glucose, triglycerides, HDL-C, and BMI, which are widely available at the primary care level. Given its determinants, METS-IR correlates with the pathophysiological components of metabolic syndrome, such as obesity and dyslipidemia.

Understanding the relationships between cardiometabolic risk factors and CRF metrics measured through CPET can provide insight into early cardiovascular dysfunction before clinical CVD is established. Furthermore, early identification of these impairments could inform preventive strategies to reduce the progression to clinical CVD. Thus, in this study, we aimed to evaluate the influence of weight, body composition, and metabolic alterations associated with insulin resistance on the cardiovascular and metabolic responses during a CPET in a cohort of subjects without established CVD.

## Methods

### Study design and recruitment

In this cross-sectional study of a single cohort of patients recruited between 4 April 2023, and 1 July 2025. Subjects were recruited through an open invitation on social media and referrals from the Endocrinology outpatient clinic of a tertiary-level hospital in Mexico City. Female and male subjects aged between 18 and 65 years were included.

Exclusion criteria were previously diagnosed pulmonary or cardiovascular disease, pregnancy, severely uncontrolled diabetes (i.e. HbA1C ≥ 10%), history of back, hip, knee, or foot pathology that hindered walking or jogging on a treadmill, chronic kidney disease with an estimated glomerular filtration rate < 60 mL/min/1.73 m^2^, and hospitalization within 3 months of the visit. The study was designed in accordance with the Declaration of Helsinki. All procedures were approved by the INCMNSZ Institutional Review Board and Ethics Committee (UIE 4497–23–23–1, ref. 4497), and all participants provided written informed consent.

### Visit summary

A patient history of cardiovascular disease, diabetes, hypertension, dyslipidemia, current medications, tobacco, and alcohol use was obtained. Diabetes status was documented through self-report, medical records from the outpatient endocrinology clinic, or with biochemical measurements obtained within 3 months of the visit (fasting glucose >126 mg/dL and/or HbA1c > 6.5%). The level of physical activity was assessed through the International Physical Activity Questionnaire 9 (IPAQ-9).^24^ Height and weight were obtained for all subjects, and a subset of 386 (81%) subjects underwent comprehensive body composition analysis. Indirect calorimetry at rest and a fasting blood sample were obtained from 356 (75%) subjects. All subjects underwent CPET.

### Body composition analysis

Subject height was obtained using a floor stadiometer (SECA). Waist and hip circumferences were obtained using an anthropometric measuring tape. Body composition was assessed via bioelectrical impedance analysis (BIA) with a SECA mBCA514 medical body composition analyzer. BIA analysis included: weight (kg), fat mass (kg and %, FM), fat-free mass (kg and %, FFM), skeletal muscle mass (kg, SMM), total body water (TBW %), visceral adipose tissue (VAT, L), and phase angle (°). The fat-free mass to fat mass ratio (FFM/FM) was calculated by dividing the percentage of fat-free mass by fat mass. Body mass index (BMI) was calculated using the standard formula, where weight is in kg and height in m^2,^ and normal weight (BMI 18.5–24.9 kg/m^2^), overweight (25–29.9 kg/m^2^), and obesity (≥ 30 kg/m^2^) were defined according to the recommendations by the World Health Organization.^25^

### Insulin sensitivity assessment

Insulin sensitivity was assessed using four validated equations: the metabolic score insulin resistance index (METS-IR), the metabolic score for visceral fat (METS-VF), the homeostasis model assessment – insulin resistance (HOMA-IR), and the quantitative insulin sensitivity check index (QUICKI).^21–23,26^ HOMA and QUICKI, which require insulin measurement, were used in 356 subjects. METS-IR and METS-VF were used in 402 subjects.

METS-IR was calculated as advised by its developers^23^:


(1) $$\mathrm{METS}-\mathrm{IR}=\frac{\left[\ln(2\times G0+TG0)\times \mathrm{BMI}\right]}{\left[\ln(\mathrm{HDL}-C)\right]}$$

where G0 is fasting glucose in mg/dL, TG0 fasting triglycerides in mg/dL, and HDL-C is high-density lipoprotein cholesterol in mg/dL. METS-IR was developed as a surrogate for FFM-adjusted M value in a hyperinsulinemic euglycemic clamp. The cut-off for insulin resistance was ≥ 50 kg/m^2^.

METS-VF was calculated as^26^:


(2) $$\mathrm{METS}-\mathrm{VF}=4.466+0.011\times [\ln(\mathrm{METS}-\mathrm{IR}){]}^{3}+3.239\times [\ln(\mathrm{WHtr}){]}^{3}+0.319\times (\mathrm{Sex})+0.594\times [\ln(\mathrm{Age})]$$

where WHtr corresponds to the weight-to-height ratio, sex equals 1 for males and 0 for females, and age is in years. The cut-off for insulin resistance was > 7.18.

HOMA-IR was calculated as^22^


(3) $$\mathrm{HOMA}-\mathrm{IR}=\frac{\left[G0\times \mathrm{insulin}\right]}{405}\;$$

where the fasting insulin is in µU/mL and glucose in mg/dL, it is worth highlighting that HOMA-IR has been validated against the unadjusted M value from a hyperinsulinaemic euglycemic clamp. The cut-off for insulin resistance was ≥ 2.5

QUICKI was calculated as^21^


(4) $$\mathrm{QUICKI}=\frac{1}{\left[\log(\mathrm{insulin})+\log(G0)\right]}\;,$$

where G0: fasting glucose in mg/dL, TG0: fasting triglycerides in mg/dL. The cut-off for insulin resistance was ≤ 0.35

### Cardiopulmonary exercise test

CPET was performed using a Cardiovit CS-200 Ergo-Spiro stress test system (Schiller) coupled to a motorized treadmill (TMX 428, Schiller) and gas analyzer (PowerCube, Ganshorn Medizin Electronic). The system was calibrated daily for atmospheric conditions using a thermohydrometer, volumes using a 2L syringe, and gases with a gas mixture composed of 5% carbon dioxide (CO_2_), 16% oxygen (O_2_), and 79% nitrogen (N_2_). Baseline 12-lead ECG and spirometry were obtained for all subjects to screen for arrhythmia or respiratory pathology before starting the test. Traditional or modified Bruce protocols^27^ were used for subjects with BMI < 35 kg/m^2^ and BMI ≥ 35 kg/m^2^, respectively. The protocol included a 40-second active rest phase for breath stabilization. Participants without previous treadmill experience were given a one-minute stabilization walking phase at 0.5 km/h and 0% incline. The traditional Bruce protocol consists of 7 incremental 3-min stages: (1) 2.7 km/h, 10% incline, 4.7 METS; (2) 4.0 km/h, 12% incline, 6.8 METS; (3) 5.4 km/h, 14% incline, 9.1 METS; (4) 6.7 km/h, 16%, 12.9 METS; (5) 8.0 km/h, 18% incline, 15 METS; (6) 8.8 km/h, 20% incline, 16.9 METS; and (7) 9.6 km/h, 22% incline, 19.1 METS. The modified Bruce protocol starts with two additional lower intensity 3-min stages: (1) 2.7 km/h, 0%, 2.9 METS; and (2) 2.7 km/h, 5%, 3.7 METS, followed by the traditional Bruce protocol stages.

Throughout CPET, subjects were monitored via continuous 12-lead ECG and automatic blood pressure measurements at 3-min intervals (BP-200 plus, Schiller). The rate of perceived exertion was assessed with the modified Borg scale at 1-min intervals. Active use of the treadmill handrails was not allowed unless the participant showed an unstable gait. CPET was terminated upon subject request due to exhaustion or at the physician’s discretion based on ECG changes indicative of arrhythmia or myocardial ischaemia, a drop in systolic blood pressure ≥ 20 mmHg, loss of coordination, or any other indication that subject safety could be compromised. Once maximum exertion was achieved, the test concluded with a 5-min recovery stage (i.e. a 2 km/h walk and 0% incline).

Cardiovascular, respiratory, and metabolic variables at four stages (i.e. rest, ventilatory threshold, peak exercise, and recovery) were automatically calculated by the LF8 software (version 8.5 M SR3; Ganshorn Medizin Electronic). The anaerobic threshold was calculated with the V-slope method defined by a computerized linear regression analysis of the CO_2_ output flow plotted as a function of oxygen uptake, which is thought to detect the beginning of excess CO_2_ output generated from the buffering of H^+^ arising from lactic acid​.^28^ Maximum oxygen consumption (VO_2max_) was determined when oxygen consumption no longer increased despite increasing the workload (i.e. a plateau was reached). If a plateau was not reached, the VO_2max_ was determined as the oxygen consumption at the point of maximum effort (also known as VO2peak). Wasserman plots were monitored throughout CPET. Only tests with the maximal effort criterion, defined as a respiratory RER at peak exercise ≥ 1.0, were included in the analysis^29,30^

### Indirect calorimetry

A fasting period of 4–5 h was mandatory before calorimetry. Subjects were fitted with a hermetic mask coupled to a pneumotachograph (Ganshorn Medizin Electronic). Heart rate was monitored via a 12-lead electrocardiogram (ECG) with electrodes placed in the Mason and Likar distribution. Subjects were placed in a supine position in a room with minimal visual and auditory stimuli. Following a rest phase of 20–30 min to achieve a basal state, VO2, VCO2, and heart rate were recorded at 10-second intervals for 20 min using the same gas analyzer as for CPET (PowerCube, Ganshorn Medizin Electronic). Environmental conditions, gas, and volume were calibrated daily as previously described for CPET. Resting energy expenditure (REE; kcal/day) was calculated using the Weir equation^31^


(5) REE=(1.106×VCO2+3.941×VO2)×1.44

The mean VCO_2_ and VO_2_ (mL/min) were used to calculate the respiratory quotient RQ as:


(6) $$\mathrm{RQ}\;=\;\frac{\mathrm{VCO}2}{\mathrm{VO}2}$$

Statistical and mathematical analysis

For the descriptive analysis, data normality was assessed using the Shapiro–Wilk test for skewness and kurtosis for all variables. Data are presented according to their distribution, with means and standard deviations (SD) or medians and interquartile ranges (25–75). Categorical variables are expressed as percentages.

In preparation for non-linearity assessments. Missing values were imputed using a random forest algorithm using default settings for the R function missForest::missForest. The algorithm converged after six iterations, and all out-of-bag errors fell within clinically equivalent ranges. Only predictor variables were imputed; the outcome variable (VO_2max_) was never imputed. Variables with more than 20% missigness were excluded from data imputation.

Principal component analysis (PCA) was used to identify candidate determinants of VO₂max and to guide variable selection for subsequent regression models. Input variables included clinical, metabolic, and anthropometric measures from the exercise test. The Spearman correlation matrix was computed, which inherently rank transforms the data and thereby standardizes variables to a common scale. This matrix was then diagonalized to obtain principal components—orthogonal linear combinations of the original variables ordered by explained variance.^32^ Variables with high loadings on the leading components were considered to have greater explanatory value, while variables loading on the same component were identified as potentially redundant. To facilitate interpretation, the principal component space was translated (without rotation) to center on VO₂max and ventricular response. This approach allowed data-driven selection of informative, minimally collinear predictors for inclusion in regression models, where our prespecified hypothesis was that surrogate indices of insulin resistance would predict VO₂max.

To confirm the determinants, multivariate regressions (using minimum square linear and logistic models) were performed between parameters. Given the high correlation between METS-IR and BMI, which could confound the association with VO₂max, we additionally examined BMI-adjusted residuals of METS-IR, derived from sex-specific linear regressions of METS-IR on BMI. Because insulin resistance is physiologically linked to adiposity, applying this adjustment to METS-IR alone would bias comparisons with other surrogate indices. Therefore, we computed BMI-adjusted residuals for QUICKI and HOMA-IR as well, allowing us to isolate the adiposity-independent component of insulin resistance across all indices (see Supplementary material online, Figure 3). Additionally, second-degree polynomial models for all insulin resistance markers were compared with their linear equivalents using an *F*-test and the *R* function performance::compare_performance.

Participants were grouped into tertiles 1–3 (T1-T3) based on the VO₂max levels. Multinomial logistic regression models were performed to explore the association between VO_2max_ levels and insulin resistance, and three models were fitted. Model 1 was adjusted for age (continuous) and sex. Model 2 was further adjusted for body mass index, and Model 3 was further adjusted for fat mass and physical activity levels (continuously encoded). The relative risk ratio (RRR) with 95% confidence intervals was reported. Finally, to confirm the influence of the insulin-resistant status, participants were grouped according to insulin-resistant status and differences in VO₂max and oxygen pulse (PuO₂max) were adjusted by age, gender, and BMI with ANCOVA analysis. For all inferential analyses, a *P*-value ≤ 0.05 was considered statistically significant. All analyses were performed using STATA version 15.0 and OriginPro 2024.

## Results

A total of 503 subjects were recruited. Twenty-nine (6% of the total sample) were excluded because they did not meet the maximum effort criteria RER >1. Of the 474 included in the analysis, 301 were female (64%) and 173 (36%) were male (see Supplementary material online, *F*igure  *S1*).

The cohort had a median age of 41 years (interquartile range, 26–52 years). The median body mass index (BMI) was 26.1 (23.4–30.1) kg/m², and the distribution by BMI categories was as follows: 41% normal weight, 33% overweight, and 26% obese. In terms of glucose metabolism, 18% had diabetes, 22% had prediabetes, and 60% had normal glucose levels. The distribution of these parameters was similar in both sexes.

The mean HbA1c was 5.5% (5.2–5–9), 37 (33–41 mmol/mol) in females and 5.5% (5.3–6), 37(34–42 mmol/mol) in males. The proportion of subjects with insulin resistance varied depending on the method used: METS-IR identified 22% of subjects with insulin resistance, while HOMA-IR and QUICKI identified 32% and 45%, respectively. Our primary metric for insulin resistance was QUICKI. The agreement and Kappa for QUICKI and HOMA were optimal as expected since both equations utilize the same variables for their calculation (agreement 86.6% and Kappa 0.72). The Agreement and Kappa decrease for METS-IR and QUICKI or HOMA because METS-IR do not utilize insulin and includes metrics from lipide profile and body mass index. (HOMA-METSIR = agreement 79% and Kappa 0.47, QUIKI-METSIR = agreement 70% and Kappa 0.37). The rest of biochemical panel and data of resting indirect calorimetry are presented in *Table 1*.

**Table 1:** Baseline characteristics of subjects

	Total (*n* = 474)	Females (*n* = 301)	Males (*n* = 173)
Age (years)	41 (26–52)	41 (26–53)	40 (27–51)
Normal weight (%)	41	46	34
Overweight (%)	33	28	43
Obese (%)	26	26	23
No diabetes (%)	60	61	58
Prediabetes (%)	22	20	26
Diabetes (%)	18	10	17
SBP (mmHg)	113 (103–122)	110 (100–121)	116 (107–124)
DBP (mmHg)	72 (66–78)	72 (65–78)	73 (68–79)
METS/week*	1800 (810–3210)	1637 (777–2880)	2139 (1130–3690)
Low (%)	18	21	12
Moderate (%)	54	55	54
High (%)	28	24	34
Metabolic panel			
Fasting glucose (mg/dL)	93 (86–102)	91 (85–100)	96 (88–105)
HbA1c (%)	5.5 (5.2–5.9)	5.5 (5.2–5.9)	5.5 (5.3–6.0)
Triglycerides (mg/dL)	118 (81–174)	107 (77–161)	140 (93–211)
Total cholesterol (mg/dL)	180 (152–208)	181 (152–209)	180 (151–208)
HDL-c (mg/dL)	47 (40–57)	51 (41–60)	42 (35–50)
LDL-c (mg/dL)	105 (84–133)	105 (83–133)	106 (84–133)
AST (UI/L)	21 (17–26)	20 (16–25)	23 (19–28)
ALT (UI/L)	20 (15–28)	19 (14–25)	24 (19 −33)
GGT (UI/L)	17 (12–26)	16 (12–24)	20 (14–30)
ApoB (mg/dL)	95 ± 27	94 ± 26	99 ± 28
Creatinine (mg/dL)	0.78 (0.69–0.92)	0.73 (0.64–0.81)	0.96 (0.85–1.08)
TG/glucose index	8.6 (8.2–9.1)	8.5 (8.1–9)	8.8 (8.4–9.3)
TG/HDL-c index	2.4 (1.5–4.2)	2.1 (1.3–3.7)	3.3 (2.1–5.5)
eGFR (mL/min/1.73 m^2^)	103 (90–113)	104 (93–114)	100 (85–111)
Insulin (µU/mL)	7.5 (4.9–11.9)	7.6 (5–11.8)	7.3 (4.6–12.1)
HOMA-IR	1.7 (1.1–3.1)	1.7 (1.1–2.9)	1.09 (1.1–3.1)
QUICKI	0.35 (0.32–0.38)	0.35 (0.32–0.38)	0.34 (0.32–0.38)
METS-IR	40.2 (32.7–48.8)	38.8 (31.4–47.4)	42.4 (36.4–50.6)
METS-VF	5.5 (4.1–6.5)	5 (3.7–6.2)	6.2 (5.3–6.9)
Resting indirect calorimetry			
REE (kcal/day)	1554 (1328–1869)	1425 (1231–1644)	1857 (1617–2079)
RQ	0.79 (0.77–0.82)	0.79 (0.77–0.82)	0.79 (0.76–0.82)
HR (bpm)	64 (57–70)	64 (57–70)	63 (57–70)

Values are expressed as median (IQ range 25–75) or mean ± SD.

SBP, systolic blood pressure; DBP, diastolic blood pressure; METS, metabolic equivalent = 3.5 mL O_2_/kg/min assessed by IPAQ9; BMI, body mass index; FFM, fat-free mass; FFMI, fat-free mass index; FM, fat mass; FMI, fat mass index; SMM, skeletal muscle mass; HDL-c, high density lipoprotein cholesterol; LDL-c, low density lipoprotein cholesterol calculated using the Friedwald equation; AST, aspartate transaminase; ALT, alanine transaminase; GGT, gamma-glutamyltransferase; ApoB, apolipoprotein B; TG/G, triglyceride-to-glucose index; TG/HDL, triglycerides-to-high density lipoprotein index; eGFR, estimated glomerular filtration rate by CKDEPI 2021 formula; HOMA-IR, homeostasis model assessment—insulin resistance; QUICKI, quantitative insulin sensitivity check index; METS-IR, metabolic score insulin resistance index; METS-VF, metabolic score for visceral fat; REE, resting energy expenditure; RQ, respiratory quotient; HR, heart rate (mean HR measured over 20 min).

Regarding anthropometry, BMI was 25.4 (22.7–30.5) and 26.9 (24.4–29–3) kg/m^2^; waist to hip ratio 0.84 (0.78–0.89) and 0.92 (0.88–0.99); waist to height ratio 0.53 (0.47–0.59) and 0.54 (0.50–0.59); fat mass 37.24 ± 7.8 and 26.7 ± 7.7%; FFM 62.7 ± 7.8 and 73.2 ± 7.8%; visceral fat 2 (1.2–2,6) and 3 (2.2–4.3) litres; SMM 18.2 (16.1–20.2) and 27.9 (25.1–30.1) kg; phase angle 5.15 ± 0.54 and 5.96 ± 0.55°; total body water 46.1 (42.3–50.1) and 53.1 (49.5–56.2) %, for females and males, respectively. The data are shown in *Table 2*.

**Table 2:** Anthropometry and body composition

	Total (474)	Female (301)	Male (173)
Height (cm)	163 (157–170)	159 (155–163)	172 (168–176)
Weight (kg)	71 (61–82)	66 (58–77)	79 (71–89)
BMI (kg/m^2^)	26.1 (23.4–30.1)	25.4 (22.7–30.5)	26.9 (24.4–29.3)
Waist-to-hip ratio	0.87 (0.80–0.93)	0.84 (0.78–0.89)	0.92 (0.88–0.99)
Waist-to-height ratio	0.53 (0.48–0.59)	0.53 (0.47–0.59)	0.54 (0.50–0.59)
Fat mass (kg)	23.2 (17.8–31.1)	24.3 (18.9–32.9)	21.6 (16–28.4)
Fat mass (%)	33.5 ± 9.3	37.2 ± 7.8	26.7 ± 7.7
Fat mass index	8.8 (6.6–11.7)	9.4 (7.5–13.3)	7.3 (5.7–9.9)
Fat-free-mass (kg)	45.1 (40.2–54.7)	41.7 (38.4–44.7)	58.3 (53.4–62.3)
Fat-free-mass (%)	66.4 ± 9.3	62.7 ± 7.8	73.2 ± 7.8
Fat mass index	17.4 (15.9–19.4)	16.3 (15.4–17.5)	19.8 (18.7–20.8)
FFM/FM	1.99 (1.46–2.67)	1.67 (1.31–2.17)	2.67 (2.09–3.41)
Visceral adipose tissue (L)	2.3 (1.6–3.2)	2 (1.3–2.6)	3 (2.2–4.3)
SMM (kg)	20.1 (17.3–25.9)	18.2 (16.1–20.1)	27.9 (25.1–30.1)
Phase angle (°)	5.4 ± 0.67	5.15 ± 0.54	5.96 ± 0.55
Total body water (%)	48.7 (44.1–53)	46.1 (42.3–50.1)	53.1 (49.5–56.2)

Values are expressed as median (IQ range 25–75) or mean and SD.

BMI, body mass index; FFM/FM, fat-free-mass/fat mass; SMM, skeletal muscle mass.

CPET results at rest, AT, maximum effort, and recovery stages are presented by sex to account for known differences between sexes. The effort time was 9.4 (7.5–10.4) and 11 (9.6–12.−5) minutes; the heart rate at maximum effort was 170 (157–181) and 175 (161–186) bpm, for female and male, respectively. The maximum METS were 8.5 (6.8–10.2) and 10.4 (8.7–12.−4) for females and males. Maximum oxygen consumption was 29.8 (24–36) and 36.6 (30.6–43.3) mL/kg/min. Maximum oxygen pulse adjusted by weight was 0.18 (0.15–0.21) and 0.21 (0.18–0.24) mL/kg/beat. The RER was 1.1 (1.06–1.15) and 1.13 (1.09–1.21). The ventilatory efficiency (VE/VCO2) was 28 (24.9–31.4) and 25.8 (22.9–29.3) for females and males, respectively. The indirect calorimetry during de CPET showed a glucose oxidation at maximum effort of 13 (9.7–16) and 17 (13.2–20.7) kcal/kg/h for females and males, respectively. Data of the results during CPET trajectory (active resting, anaerobic threshold and maximum effort) are shown in *Table 3*.

**Table 3:** Cardiovascular, metabolic, and ventilatory response during CPET

	Total (*n* = 474)	Female (*n* = 301)	Male (*n* = 173)
Effort time (min)	10.3 (8.3–11.3)	9.4 (7.5–10.4)	11 (9.6–12.5)
HR—active rest (bpm)	83 (73–94)	83 (73–95)	81 (74–92)
HR—AT (bpm)	131 (118–145)	128 (116–142)	133 (120–147)
HR—max (bpm)	171 (158–182)	170 (157–181)	175 (161–186)
HR Rec (bpm)	128 (115–140)	125 (111–138)	107 (101–112)
HR reserve (bpm)	107 (96–119)	106 (96–117)	112 (97–123)
METs Pred	8.5 (6.9–10.3)	7.8 (6.5–9.5)	10.3 (9.1–12.5)
METs Max	9.2 (7.5–11)	8.5 (6.8–10.2)	10.4 (8.7–12.4)
Kcal	102 (72–141)	85 (64–106)	153 (115–192)
VO_2_—Rest (mL/kg/min)	4.7 (3.7–6.1)	4.5 (3.6–5.8)	5.1 (3.9–6.6)
VO2—AT (mL/kg/min)	22 (18.5–26)	21 (17–24)	25 (21–29)
VO_2_—Max (mL/kg/min)	32.3 (26–39)	29.8 (24–36)	36.6 (30.6–43.3)
VO_2_—Rec (mL/kg/min)	18.6 (15.8–21.1)	17.6 (14.9–19.8)	20.6 (17.9–22.6)
% VO_2_—AT	70 (64–75)	70 (64–76)	68 (63–74)
PuO_2_—Rest (mL/kg/beat)	0.057 (0.044–0.072)	0.054 (0.042–0.067)	0.063 (0.048–0.080)
PuO_2_—AT (mL/kg/beat)	0.16 (0.14–0.19)	0.15 (0.13–0.18)	0.18 (0.16–0.21)
PuO_2_—Max (mL/kg/beat)	0.19 (0.19–0.22)	0.18 (0.15–0.21)	0.21 (0.18–0.24)
PuO_2_—Rec (mL/kg/beat)	0.14 (0.12–0.17)	0.13 (0.12–0.16)	0.15 (0.13–0.18)
RER—Max	1.11 (1.07–1.17)	1.10 (1.06–1.15)	1.13 (1.09–1.21)
VE/VCO_2_	27 (24.3–30.6)	28 (24.9–31.4)	25.8 (22.9–29.3)
BR—Rest (%)	89 (85–92)	89 (85–91)	89 (85–92)
BR—Max (%)	24 (13–33)	27 (15–35)	20 (11–29)
Indirect calorimetry during exercise			
Gluc ox—rest (kcal/h/kg)	0.39 (0.24–0.65)	0.39 (0.26–0.64)	0.39 (0.18–0.68)
Gluc ox—AT (kcal/h/kg)	3.3 (2.2–4.8)	2.9 (1.8–4.3)	4.3 (2.7–6.04)
Gluc ox—Max (kcal/h/kg)	14 (10.8–17.8)	13 (9.7–16)	17.3 (13.2–20.7)
Gluc ox—Rec (kcal/h/kg)	11.6 (9.2–13.5)	10.7 (8.5–12.7)	13 (11.1–14.7)

Values are expressed as median (IQ range 25–75). Rest, refers to active rest, with the subject standing on treadmill prior to the start of exercise; HR, heart rate; AT, anaerobic threshold; Pred, predicted values by Wasserman formula; Rec, recovery; METS, metabolic equivalent = 3.5 mL O_2_/kg/min; VO_2_, oxygen consumption; PuO_2_, oxygen pulse; RER, respiratory exchange rate; VE, minute ventilation; VCO_2_, carbon dioxide production; VE/VCO_2_, ventilatory efficiency; BR, breathing reserve; Glu-ox, glucose oxidation.

PCA showed that the main principal component is related to insulin resistance (28% of the variance in both males and females). While the second principal component is related to ventilatory efficiency and energy expenditure variables at maximum effort and recovery (15% and 13% of the variance in males and females, respectively). Here, within each sex, individuals with insulin resistance have a lower VO₂max than those without (see supplementary material online, *Figure S2*).

**Figure 1:**
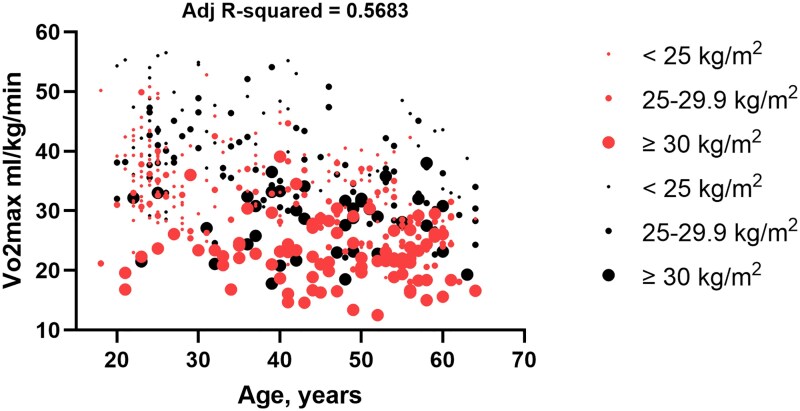
Cardiopulmonary exercise test and VO_2max_ across age, sex, and VO_2max_ categories. Red circles: females, black circles: males. 0.56 of VO_2max_ variability is explained by sex, age, and body mass index.

Logistic regression analysis revealed that VO₂max was significatively influenced by age (−0.29 mL/min/kg per year for females and −0.27 mL/min/kg per year for males). The change in VO₂max was primarily explained by sex and BMI and the adjusted R-squared for sex, age, and BMI was 0.56. Throughout all ages, males had a VO₂max of approximately 6.7 mL/kg/min higher than age-paired females. Notably, subjects with a greater BMI were concentrated at lower VO₂max values (*Figure 1*). Age-adjusted stratified analysis by BMI and sex revealed a significant decreasing gradient effect in VO₂max, PuO₂max, METs, and effort time across BMI categories (see Supplementary material online, *Table S1*). The regression models adjusted by age and divided by sex, showed that BMI explained up to 0.53 and 0.50 (adjusted *R*²) of VO₂max variability, with coefficients of −0.75 and −1.22 for females and males, respectively. Metrics associated with high body mass index, such as fat mass, FFM/FM, waist to hip ratio, and height to hip ratio, had a high biological correlation with BMI and were inversely correlated with VO₂max. HOMA, QUICKI, and METS-IR explained between 31% to 60% of VO₂variability (see Supplementary material online, *Table S2* and *S3*). Specifically, QUICKI had an R-square of 0.41 (CIs 69–109) (*P* = 0.000), 0.44 (Cis 79–139) (*P* = 0.000), HOMA 0.31 (CIs-1.4− −0.65) (*P* = 0.000), 0.38 (CIs-2.73− −1.34) (*P* = 0.000), for women and men, respectively.

The analysis of VO₂max terciles showed that individuals in the lowest tercile were older, had a higher body mass index, higher insulin resistance index, and lower carbohydrate oxidation during maximal exercise in the CPET test, compared to the other two terciles (*Table 4*). The multinomial logistic regression where VO₂max divided into terciles was the dependent variable (second tercile as reference) showed that after adjusting for sex and age (model 1), QUICKI insulin resistance index was associated with a significant increase in the relative risk ratio of 2.46 (1.4–4.3) of belonging to the lowest tercile and decrease the probability of belonging to the highest tertile. After adjusting for model 1 plus BMI (model 2) and model 2 plus fat mass % and physical activity level (model 3), the model showed a significant reduction in belonging to the highest tertile (0.35, 0.14–0.86) (see Supplementary material online, table  *S5*). These results were replicated when HOMA and METS-IR were included as the main independent variables.

**Table 4:** VO_2_ max divided into tertiles

N 301	Tertile 1 *n* = 159	Tertile 2 *n* = 158	Tertile 3 *n* = 157	*p*
VO_2max_ mL/kg/min	23.4 (21–26.3)	32.4 (30.4–34.3)	41.2 (38.6–46.3)	
Female *n* (%)	130 (82)	102 (65)	69 (44)	0.000
Age	50 (40–56)	40 (26–51)	30 (24–41)	0.0001
BMI	30.7 (27.1.6–34.7)	25.4 (23.4–29.0)	23.8 (21.6–26.1)	0.0001
FM %	42.6 (37.2–46)	33.5 (30.3–37.5)	26 (21–30)	0.0001
FFM %	57.4 (54–63)	66.5 (62.5–69.7)	74 (70–79.5)	0.0001
TBW %	42.8 (40.5–46.3)	48.8 (45.9–51)	53.9 (50.9–57.6)	0.0001
PA °	5.2 ± 0.65	5.4 ± 0.59	5.7 ± 0.67	0.0000
METS-IR	48.2 (40.7–57.3)	38.7 (32.4–46.7)	33.4 (30.3–38.2)	0.0001
HOMA	2.7 (1.7–4)	1.8 (1.2–2.9)	0.95 (0.75–1.6)	0.0001
QUICKI	0.33 (0.31–0.35)	0.35 (0.33–0.37)	0.4 (0.36–0.40)	0.0001
CHO_max_	692 (584–833)	934 (773–1151)	1264 (958–1510)	0.0001

Values are expressed as median (IQ range 25–75).

VO_2_, oxygen consumption; HOMA-IR, homeostasis model assessment—insulin resistance; QUICKI, quantitative insulin sensitivity check index; METS-IR, metabolic score insulin resistance index; BMI, body mass index; FFM, fat-free mass; TBW, total body water; FM, fat mass; SMM, skeletal muscle mass; PA, phase angle; SMM, skeletal muscle mass; CHO_max_: maximum carbohydrate oxidation.

**Figure 2:**
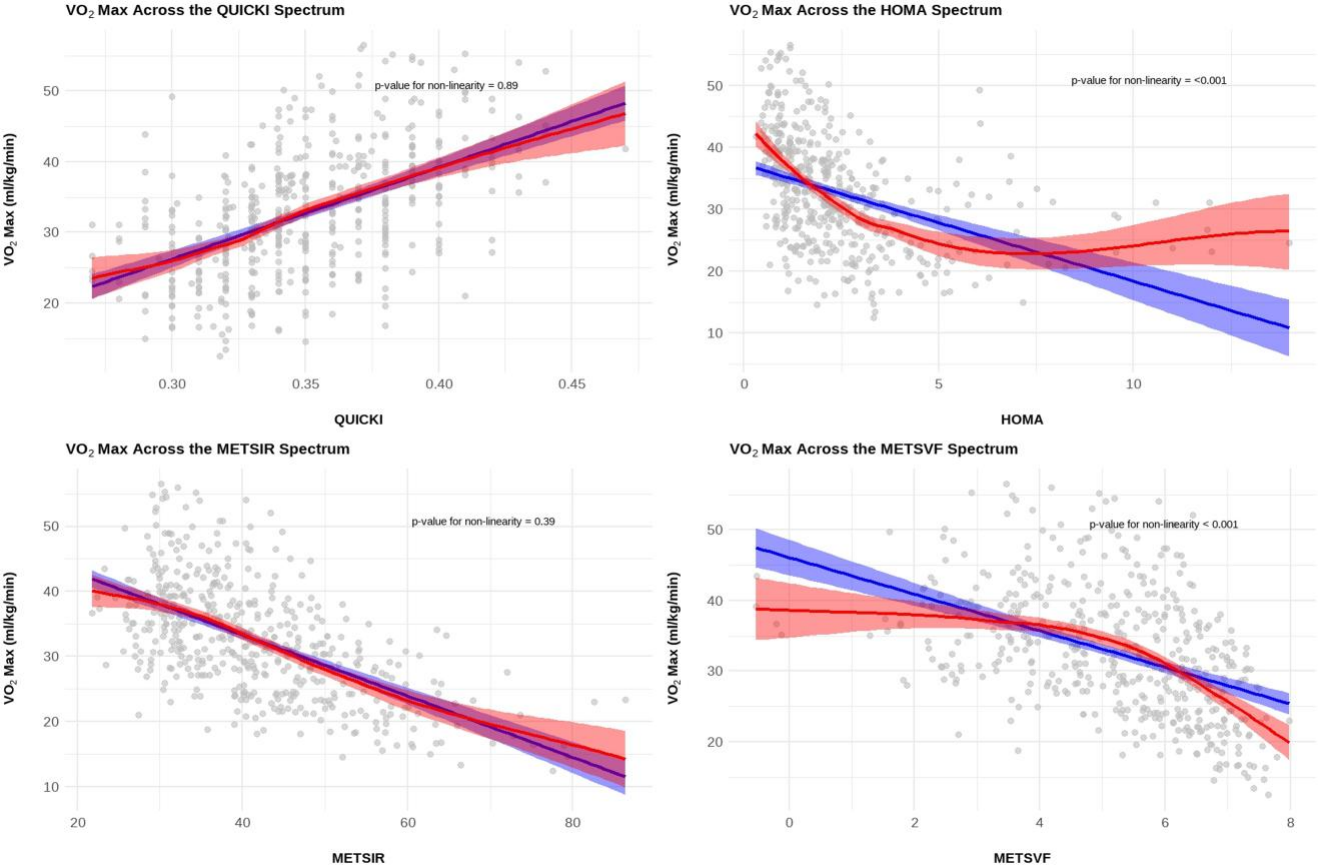
VO_2max_ across insulin resistance. Scatter plots of the relationship of VO_2max_ among different indices of insulin resistance. Shaded areas indicate 95% confidence intervals. The quadratic models capture the curvature in the data that linear models fail to accommodate, particularly at higher levels of insulin resistance.

*F*-tests indicated that quadratic models provided significantly better fit than linear models for most insulin resistance indices when predicting VO₂max from cardiopulmonary exercise testing (*Figure 2*). The sign of the quadratic coefficient offers insight into the underlying pathophysiology: negative coefficients suggest threshold effects, where the deleterious impact of insulin resistance on cardiovascular fitness accelerates beyond a critical level, consistent with the activation of additional pathological pathways accompanying metabolic disease onset. Positive coefficients indicate saturation, where the negative effect of worsening insulin resistance on fitness attenuates at higher levels. QUICKI and METS-IR did not exhibit significant non-linearity, likely because their logarithmic transformations already capture the curvilinear relationship.

Finally, participants with measured insulin were subanalyzed to assess the effect of insulin resistance on CPET performance. Insulin resistance was defined as QUICKI <0.35 and/or HOMA-IR >2.5. As BMI was shown to be an important predictor of VO_2max_ in PCA, and METS-IR includes BMI in its components, METS-IR was not considered in this subanalysis. VO₂max and PuO₂max were 20% lower in subjects with IR than in those without (see Supplementary material online, *Table S4*). Notably, these differences persisted after adjustment for BMI, underscoring the independent effect of IR on CPET performance.

## Discussion

Cardiorespiratory fitness, primarily defined by VO₂max, is influenced by biological factors such as age and sex.^30,33,34^ In addition, cardiometabolic risk factors associated with insulin resistance, such as obesity, dyslipidemia, and prediabetes/diabetes, may affect VO₂max prematurely and have potentially deleterious implications in all-cause and cardiovascular mortality.^13,15,35,36^ The purpose of this study was to evaluate the influence of weight, body composition, and metabolic alterations associated with insulin resistance on the cardiovascular and metabolic response during a CPET. We found that VO₂max was determined by BMI, fat-free mass, and insulin resistance independently of age and sex.

We studied a single cohort of individuals without established CVD via graded CPET with the Bruce protocol. The Bruce protocol is a widely validated treadmill test frequently employed in both hospital and research settings for graded exercise assessments.^37–39^ It measures physiological responses to exercise and, when coupled with indirect calorimetry, provides a direct evaluation of VO₂max. VO₂max assessment via the Bruce protocol is comparable to other protocols, including the Balke, Ellestad, and Astrand protocols.^40,41^

The PCA and logistic regression analyses showed that BMI, FFM %, FM % and QUICKI were the main determinants of VO₂max. Notably, variables in biological collinearity with these metrics also exhibited a strong association with VO₂max, which reinforced our findings. The strong association between VO₂max reduction and obesity reported herein is in line with previous findings.^10,15,42–44^ The most robust study of CPET in individuals with obesity included 1594 participants (755 obese). It demonstrated a difference in VO₂max of 14 mL/kg/min between the lean and obese groups, with a gradient effect among obesity classes. Hunlens *et al*. showed a difference in VO₂max between obese and lean women (BMI 38 vs. 22 kg/m^2^) of 10.3 mL/kg/min (*P* = 0.0001).^45^ Other studies in adolescents and children have yielded similar results.^43,46^ In our study, the difference between the lean and obese groups was 13 mL/kg/min. We assessed obesity as a single group, as the small number of individuals with morbid obesity (*n* = 13) prevented comparisons between obesity classes with sufficient statistical power. Additionally, the very low prevalence of the metabolic healthy obesity phenotype in our cohort (*n* = 17 cases) precludes a subanalysis with enough power.

Insulin resistance, as determined by HOMA and QUICKI, which was more prevalent among individuals with obesity, showed a strong, non-linear inverse association with VO₂max. Previously, a negative correlation was described between insulin resistance determined by QUICKI and VO₂max calculated by the 6-min walk test (6MWT) in overweight and obese people (R 0.77, *P* < 0.001).^47^ Leite *et al*. reported VO₂max reduction as an early marker of insulin resistance in subjects at high risk of type 2 diabetes.^17^ Furthermore, VO_2max_ is reduced in diabetes, which was replicated in our study, reinforcing the association between insulin resistance and CRF.^11,13,17^

Insulin resistance was inversely related to the muscle-to-fat ratio. This association is well-documented in studies using the euglycemic-hyperinsulinaemic clamp test, which is considered the gold standard for assessing insulin sensitivity.^20^ Kurinami *et al*. found a correlation between the *M*/*I* value and the muscle-to-fat ratio, with a correlation coefficient of *B* = 0.806 and *P* < 0.001.^48^ Moreover, the muscle-to-fat ratio is positively correlated with CVD and mortality.^49,50^ Taken together, these findings support the hypothesis that fat mass, which represents the metabolic load, interacts with fat-free mass (muscle), which represents metabolic capacity, and imbalances between these components determine the risk of cardiometabolic diseases.^51^ Notably, in our study, METS-IR explained better the VO₂max variance (Adj *R*-squared 0.51 and 0.60 for females and males) than HOMA-IR and QUICKI, likely because it combines metabolic (glucose, HDL-C, and triglycerides) and anthropometric (BMI) parameters. To better explore the effect of insulin resistance, we selected QUICKI as the primary index, and the association was further confirmed with HOMA and METS-IR.

This work presents two closely associated quantitative assessments of metabolic health and cardiovascular fitness: insulin resistance and VO₂max. The mechanistic interrelationship between the two impacts glucose metabolism via the tricarboxylic acid cycle and oxidative phosphorylation, which are influenced by mitochondrial function.^52,53^ Albeit VO₂max is assessed in exercise and insulin resistance at rest, both measurements provide a quantitative assessment of the maximum possible uptake of molecules required for cellular respiration in response to exercise or hyperinsulinaemia. The potential mechanisms underlying the reduction in CRF include decreased relative muscle mass and insulin resistance, which reduce muscle glucose uptake and O_2_ consumption during maximum effort, where anaerobic glycolysis plays a central role.

Maximum oxygen pulse (PuO_2_) adjusted by weight had the same determinants as VO₂max, as expected in persons without advanced cardiovascular disease. Specifically, we found that PuO_2_ impairment was associated with increasing BMI, insulin resistance, and relative fat mass. Former studies did not find differences in PuO_2_ between lean and obese individuals.^36^ However, these studies did not adjust PuO_2_ by weight, potentially masking the reduction in PuO_2_ relative to body mass in their obese populations. PuO_2_, calculated as the VO_2_/HR ratio, is a surrogate of stroke volume (SV) and has been proposed as a marker of ventricular function.^54^ Given that the HR response during CPET was as expected by age, the decrease in PuO_2_ observed in our study is likely attributable to a reduction in VO_2_. Thus, individuals with low relative muscle mass and compromised metabolic health may exhibit subclinical impairment of ventricular function that becomes evident during maximum effort. Studies combining CPET with echocardiography would further support this association.^55^

Other CPET metrics important for CRF assessment are achieved METs and ventilatory efficiency. In our study, subjects with cardiometabolic risk factors, such as insulin resistance and obesity, achieved fewer METs than those without, further confirming CRF compromise. However, ventilatory efficiency was not influenced by these risk factors. This is in line with reports by others showing that ventilatory mechanics were not compromised in persons with morbid obesity.^14,15^ Interestingly, De Jon *et al*. demonstrated that, although morbidly obese subjects had VO₂max comparable to individuals with heart failure NYHA classes II–IV, the ventilatory efficiency was preserved in obesity.^14^ Taken together, these results suggest that there is independence between ventilatory mechanics and cardiometabolic risk factors.

Of course, this study also has some limitations. First, the limited number of people with morbid obesity hindered stratified analysis by obesity class, which would have further characterized the role of excess adiposity in CRF. Second, body composition was not evaluated with dual-energy X-ray absorptiometry (DEXA), which is the gold standard.^56^ However, bioelectrical impedance analysis correlates well with DEXA and is more readily available in the clinical setting.^57^ Third, insulin resistance was estimated via formulas rather than evaluated using the euglycemic-hyperinsulinaemic clamp, which is the gold standard. Notwithstanding, we employed four validated formulas to enhance the robustness of our analysis.

The study's overarching strength lies in the use of ergospirometry, the gold standard, for evaluating VO₂max. This permitted direct measurement of VO₂max rather than formula-based calculation. Although there is evidence of the association between CRF and insulin resistance, these studies have not used CPET, the gold standard to measure VO₂max, and instead rely on equations that can have limitations for CRF estimation. In addition, we employed the Bruce protocol, a standardized and widely used method in research and clinical settings, which promotes result interpretability and comparability. Moreover, the Bruce protocol allows for the calculation of VO₂max using formulas when indirect calorimetry is not available, thereby facilitating comparison with studies in the field that use other methodologies. Finally, our cohort had a high prevalence of overweight, obesity, prediabetes, and diabetes that resembles official reports of our underlying population (ENSANUT),^58^ underscoring the potential for extrapolation of our findings into the clinical setting. However, further studies with probabilistic sampling are warranted to statistically extrapolate the results to the Mexican population.

As conclusions, in a cohort of individuals without established CVD, the main determinants of CRF were BMI, fat-free mass, fat mass, and insulin resistance. A gradient effect was observed among VO_2max_ categories, independent of traditional VO₂max determinants, including age and sex. The potential mechanisms underlying the reduction in CRF are a decreased relative muscle mass available for adequate glucose uptake and O_2_ consumption when adiposity is in excess and insulin resistance is present. Our results suggest that early and asymptomatic alterations in CRF are detectable during maximum effort and are associated with an increased prevalence of insulin resistance. VO₂max is a trainable metric and insulin resistance, a modifiable risk factor for metabolic diseases, and both can be improved by lifestyle interventions. The assessment of cardiorespiratory fitness should be considered systematically in the evaluation of persons with cardiometabolic risk factors.

## Supplementary Material

oeag029_Supplementary_Data

## Data Availability

Anonymized data for research purposes is available upon request to the corresponding author.
