# Supplementary material for: Insulin resistance and metabolic health predict cardiorespiratory fitness: cohort study
Source: Eur Heart J Open. 2026 Feb 25;6(2):oeag029. doi: 10.1093/ehjopen/oeag029 (PMC12978527; doi:10.1093/ehjopen/oeag029)
Supplement: oeag029_Supplementary_Data [file oeag029_supplementary_data.docx]

**Supplementary Table 1. CPET across BMI categories by sex**

|  | **Female** | | | **Male** | | |
| --- | --- | --- | --- | --- | --- | --- |
|  | **Normal weight**  **(n = 137)** | **Overweight**  **(n = 83)** | **Obese**  **(n = 81)** | **Normal weight**  **(n = 58)** | **Overweight**  **(n = 75)** | **Obese**  **(n = 40)** |
| VO2 Rest (mL/min/kg) | 4.5  (3.8-5.6) | 4.4  (3.4-5.8) | 4.6  (3.3-5.9) | 5.7  (4.5-7.5)^#^ | 3.5  (3.5-6.9) | 4.7  (3.9-5.9)^*^ |
| VO2 AT (mL/min/kg) | 23.3  (20.1-26.55)^&#^ | 20.6  (18-23.7)^*#^ | 16.1  (13-19.3)^*&^ | 27.6  (22.8-32.3)^#^ | 25.1  (21.5-29)^#^ | 20.4  (16.8-22.7)^*&^ |
| VO2 Max (mL/min/kg) | 35  (30.7-39.4)^&#^ | 28.9  (24.5-33.5)^*#^ | 22.3  (18.9-25.8)^*&^ | 42.9  (37.9-48.5)^&#^ | 37.1  (31.3-42.4)^*#^ | 28.8  (23.2-32)^*&^ |
| VO2 Rec (mL/min/kg) | 18.8  (16.7-20.7)^#^ | 17.8  (15.5-20.2)^#^ | 14.5  (12.8-17.5)^&*^ | 22.3  (20.1-24.8)^&#^ | 20.6  (18-22.6)^*#^ | 17.8  (15.2-20.6)^*&^ |
| PuO2 Rest (mL/beat/kg) | 0.053  (0.044-0.069) | 0.056  (0.045-0.068) | 0.051  (0.039-0.066) | 0.064  (0.055-0.086) | 0.063  (0.045-0.082) | 0.059  (0.049-0.071) |
| PuO2 AT (mL/beat/kg) | 0.17  (0.15-0.19)^#^ | 0.16  (0.13-0.18)^#^ | 0.12  (0.10-0.14)^*&^ | 0.21  (0.17-0.23)^#^ | 0.18  (0.16-0.21)^#^ | 0.15  (0.13-0.18)^*&^ |
| PuO2 Max (mL/beat/kg) | 0.20  (0.17-0.23)^&#^ | 0.18  (0.15-0.19)^*#^ | 0.14  (0.12-0.16)^*&^ | 0.24  (0.21-0.28)^&#^ | 0.21  (0.18-0.24)^*#^ | 0.17  (0.14-0.20)^*&^ |
| PuO2 Rec (mL/beat/kg) | 0.14  (0.12-0.17)^#^ | 0.14  (0.10-0.16)^#^ | 0.11  (0.10-0.13)^*&^ | 0.15  (0.13-0.18) | 0.16  (0.14-0.18) | 0.14  (0.12-0.17) |
| METS Max | 10  (8.8-11.2)^&#^ | 8.3  (7-9.5)^*#^ | 6.4  (5.4-7.4)^*&^ | 12.2  (10.8-13.9)^&#^ | 10.6  (8.9-12.1)^*#^ | 8.2  (6.7-9.1)^*&^ |
| VE/VCO2 | 28.8  (24.9-32.7) | 27.7  (24.5-29.9) | 27.6  (25-30.8) | 25.5  (22.7-28.7) | 25.7  (22.9-29.1) | 26.1  (24.5-31.2) |
| Glu-ox Rest  (kcal/h/kg) | 0.42  (0.28-0.65) | 0.36  (0.24-0.69) | 0.35  (0.25-0.58) | 0.41  (0.24-0.64) | 0.38  (0.18-0.7) | 0.45  (0.14-0.68) |
| Glu-ox AT  (kcal/h/kg) | 3.1  (2.1-4.6)^&,#^ | 3.2  (1.6-4)^*^ | 2.6  (1.6-3.2)^*^ | 4.8  (2.9-7.2) | 4.3  (2.8-6.2) | 2.8  (2.2-4.7) |
| Glu-ox Max  (kcal/h/kg) | 15.2  (12.5-17) | 11.8  (9.7-15.2) | 9.0  (8.0-10.2) | 19.9  (16.7-22.8)^#^ | 17.3  (12.6-20.3)^#^ | 12.7  (10.3-13.8)^*&^ |
| Glu-ox Rec  (kcal/h/kg) | 11.6  (9.7-13.3) | 10.9  (8.8-13.1) | 7.9  (6.9-10.1) | 14.4  (12.1-16.9) | 12.9  (10.7-14.3) | 11.3  (9.1-12.6) |
| Effort time  (min) | 10.1  (9.07-11.1)^&#^ | 9  (7.3-10.2)^*^ | 7.4  (6.4-9.4)^*^ | 12.1  (11.0-13.2)^&#^ | 11.2  (10-12.5)^*#^ | 9.4  (7.5-10.3)^*&^ |

Values expressed as median (IQ range 25-75). *p*-values indicate differences between groups by Kruskal-Wallis and Dunne post-hoc test; *p* < 0.05 is considered significant. (*) indicate statistical significance compared to the normal weight group, (&) indicates statistical significance compared to the overweight group, (#) indicates significance compared to obese. VO2: oxygen consumption, Rest: subject standing on the treadmill prior to the start of exercise, AT: anaerobic threshold, Rec: recovery, PuO2: oxygen pulse, METS: metabolic equivalent = 3.5 mL O2/kg/min, Glu-ox: glucose oxidation, VE/VCO2: ventilatory efficiency

**Supplementary table 2.** VO2max regressions adjusted by age in females

|  | R-2 | coef | t | p | Lower Interval | Upper Interval |
| --- | --- | --- | --- | --- | --- | --- |
| Age | 0.23 | -0.29 | -9.75 | 0.000 | -0.35 | -0.23 |
| BMI | 0.53 | -0.75 | -13.63 | 0.000 | -0.86 | -0.64 |
| HOMA | 0.31 | -1.01 | -5.43 | 0.000 | -1.38 | -0.65 |
| QUICKI | 0.41 | 89 | 8.76 | 0.000 | 69 | 109 |
| FM (kg) | 0.58 | -0.45 | -13.07 | 0.000 | -0.51 | -0.38 |
| FM (%) | 0.56 | -0.61 | -12.59 | 0.000 | -0.71 | -0.52 |
| FMI | 0.56 | -1.09 | -12.37 | 0.000 | -1.26 | -0.92 |
| FFM (kg) | 0.37 | -0.49 | -5.68 | 0.009 | -0.54 | -0.26 |
| FFM (%) | 0.58 | 0.64 | 12.92 | 0.000 | 0.54 | 0.73 |
| FFM/FM | 0.54 | 6.97 | 11.80 | 0.000 | 5.80 | 8.13 |
| TBW (%) | 0.55 | 0.80 | 12.16 | 0.000 | 0.67 | 0.93 |
| VAT (L) | 0.43 | -2.97 | -8.05 | 0.000 | -3.7 | -2.24 |
| Waist (cm) | 0.48 | -0.29 | -9.95 | 0.000 | -0.34 | -0.23 |
| Waist-to-Hip Ratio | 0.32 | -21.86 | -4.06 | 0.000 | -32 | -11 |
| Waist-to-Height ratio | 0.42 | -32 | -7.8 | 0.000 | -40 | -23 |
| HDL-c (mg/dL) | 0.29 | 0.15 | 5.16 | 0.000 | 0.10 | 0.21 |
| HbA1c (%) | 0.26 | -1.34 | -2.45 | 0.015 | -2.42 | -0.26 |
| Glucose-TGL Index | 0.27 | -14.36 | -4.13 | 0.000 | -21 | -7.5 |
| METS-IR | 0.51 | -0.38 | -12.46 | 0.000 | -0.44 | -0.32 |
| Status HOMA | 0.36 | -6.2 | -7.08 | 0.000 | -7.92 | -4.47 |
| CHO-OX-max | 0.45 | 0.014 | 8.68 | 0.000 | 0.011 | 0.018 |
| INSULIN | 0.34 | -0.35 | -6.64 | 0.000 | -0.46 | -0.25 |

BMI: body mass index, FFM: fat-free mass, FFMI: fat-free mass index, FM: fat mass, FMI: fat mass index, HOMA: homeostasis model assessment – insulin resistance, QUICKI: quantitative insulin sensitivity check index, METS-IR: metabolic score insulin resistance index, TBW: total body water, CHO-ox, carbohydrate oxidation at maximum effort

**Supplemenary table 3**. VO2max regressions adjusted by age in males

|  | R-2 | coef | t | p | Lower Interval | Upper Interval |
| --- | --- | --- | --- | --- | --- | --- |
| Age | 0.16 | -0.27 | -5.76 | 0.000 | -0.36 | -0.18 |
| BMI | 0.50 | -1.22 | -10.97 | 0.000 | -1.44 | -1.00 |
| HOMA | 0.38 | -2.04 | -5.85 | 0.000 | -2.73 | -1.35 |
| QUICKI | 0.44 | 109 | 7.24 | 0.000 | 79 | 139 |
| FM (kg) | 0.46 | -0.61 | -10.85 | 0.000 | -0.72 | -0.5 |
| FM (%) | 0.55 | -0.72 | -10.65 | 0.000 | -0.86 | -0.59 |
| FMI | 0.57 | -1.77 | -11.15 | 0.000 | -2.08 | -1.45 |
| FFM (kg) | 0.21 | -0.23 | -2.48 | 0.014 | -0.41 | 0.05 |
| FFM (%) | 0.55 | 0.72 | 10.65 | 0.000 | 0.59 | 0.86 |
| FFM/FM | 0.35 | 1.64 | 6.03 | 0.000 | 1.1 | 2.18 |
| TBW (%) | 0.56 | 1.02 | 10.74 | 0.000 | 0.83 | 1.21 |
| VAT (L) | 0.46 | -2.91 | -8.39 | 0.000 | -3.6 | -2.23 |
| Waist (cm) | 0.56 | -0.47 | -10.65 | 0.000 | -0.56 | -0.38 |
| Waist-to-Hip ratio | 0.45 | -62.42 | -7.75 | 0.000 | -78 | -46 |
| Waist-to-Height | 0.58 | -83.84 | -11.15 | 0.000 | -98 | -68 |
| HDL-c (mg/dL) | 0.32 | 0.31 | 4.98 | 0.000 | 0.18 | 0.43 |
| HbA1c (%) | 0.23 | -1.7 | -2.1 | 0.037 | -3.28 | -0.09 |
| Glucose-TGL index | 0.32 | -22.63 | -5.12 | 0.000 | -31 | -13 |
| METS-IR | 0.60 | -0.56 | -11.57 | 0.000 | -0.65 | -0.46 |
| Status HOMA | 0.19 | -8.12 | -5.35 | 0.000 | -11.12 | -5.11 |
| CHO-OX | 0.40 | 0.012 | 6.58 | 0.000 | 0.008 | 0.016 |
| INSULIN | 0.42 | -0.66 | -6.85 | 0.000 | -0.84 | -0.47 |

BMI: body mass index, FFM: fat-free mass, FFMI: fat-free mass index, FM: fat mass, FMI: fat mass index, HOMA: homeostasis model assessment – insulin resistance, QUICKI: quantitative insulin sensitivity check index, METS-IR: metabolic score insulin resistance index. TBW: total body water

**Supplementary table 4. VO2max and PuO2 relationship with insulin resistance**

|  | Without IR  (n = 162) | IR  (n = 194) |
| --- | --- | --- |
| Age (years) | 37 (25 -51) | 45 (33 – 54) |
| BMI (kg/m^2^) | 24.1 (22.2 – 26.1) | 30.3 (26.7 – 33.6) |
| VO2max (ml/kg/min) | 35 (28.8 – 40.9) | 28.3 (22.8 – 32.5) |
| PuO2 (ml/kg/beat) | 0.2 (0.17 – 0.23) | 0.17 (0.14 – 0.19)* |

* *p* < 0.05 between IR and No-IR, adjusted by BMI and sex (ANCOVA).

IR: Insulin Resistance

**Supplementary table 5.** Multinomial logistic regression with QUICKI as main independent variable and VO2max tertiles as dependent variable

|  | Tertile 1  (21 – 26.3 ml/kg/min) | Tertile 2  (30.4 – 34.3 ml/kg/min) | Tertile 3  (38.6 – 46.3 ml/kg/min) |
| --- | --- | --- | --- |
| IR cases/total  QUICKI > 0.35 | 83/133 | 57/119 | 19/104 |
| Model 1 | 2.46 (1.4 – 4.3) | Reference | 0.18 (0.09 – 0.36)*** |
| Model 2 | 0.75 (0.4 – 1.5) | Reference | 0.31 (0.15 – 0.66)** |
| Model 3 | 1.0 (0.41 – 2.5) | Reference | 0.35 (0.14 – 0.86)* |

Model 1: adjusted by sex and age

Model 2: Adjusted by model 1 plus BMI,

Model 3: Adjusted by model 2 plus, FM%, physical activity


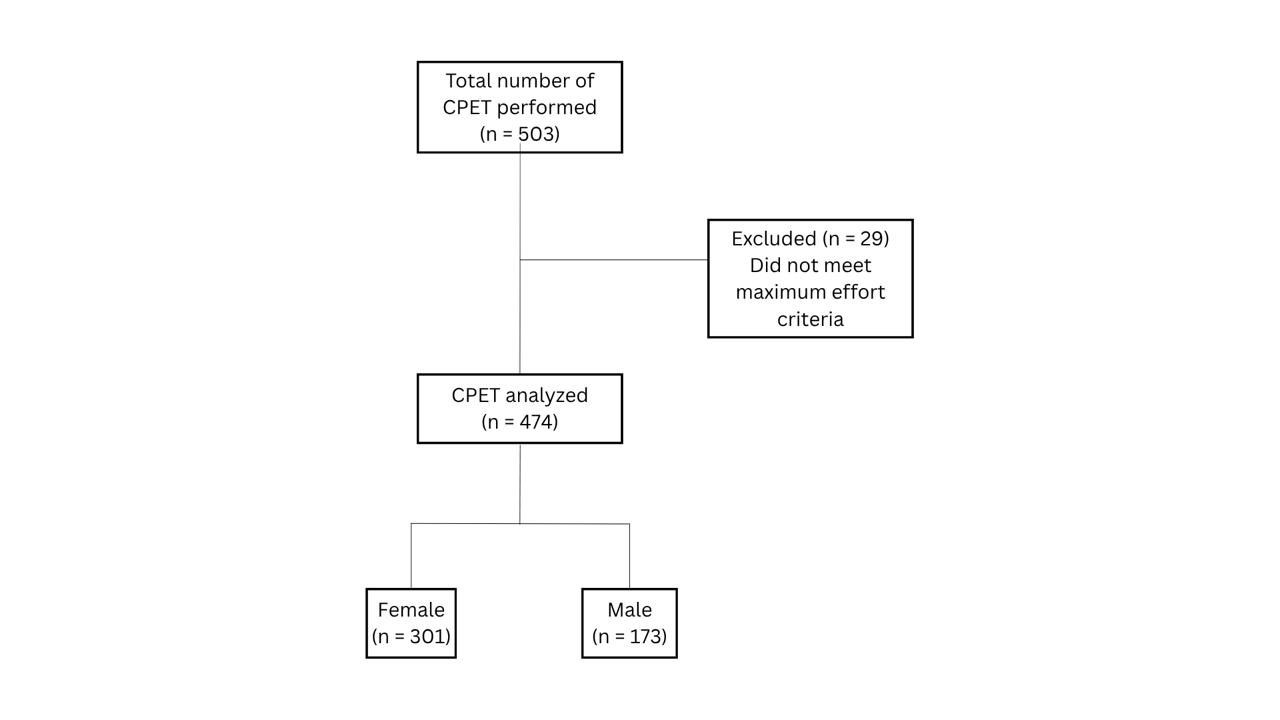


**Supp fig 1**. Flow chart of participants


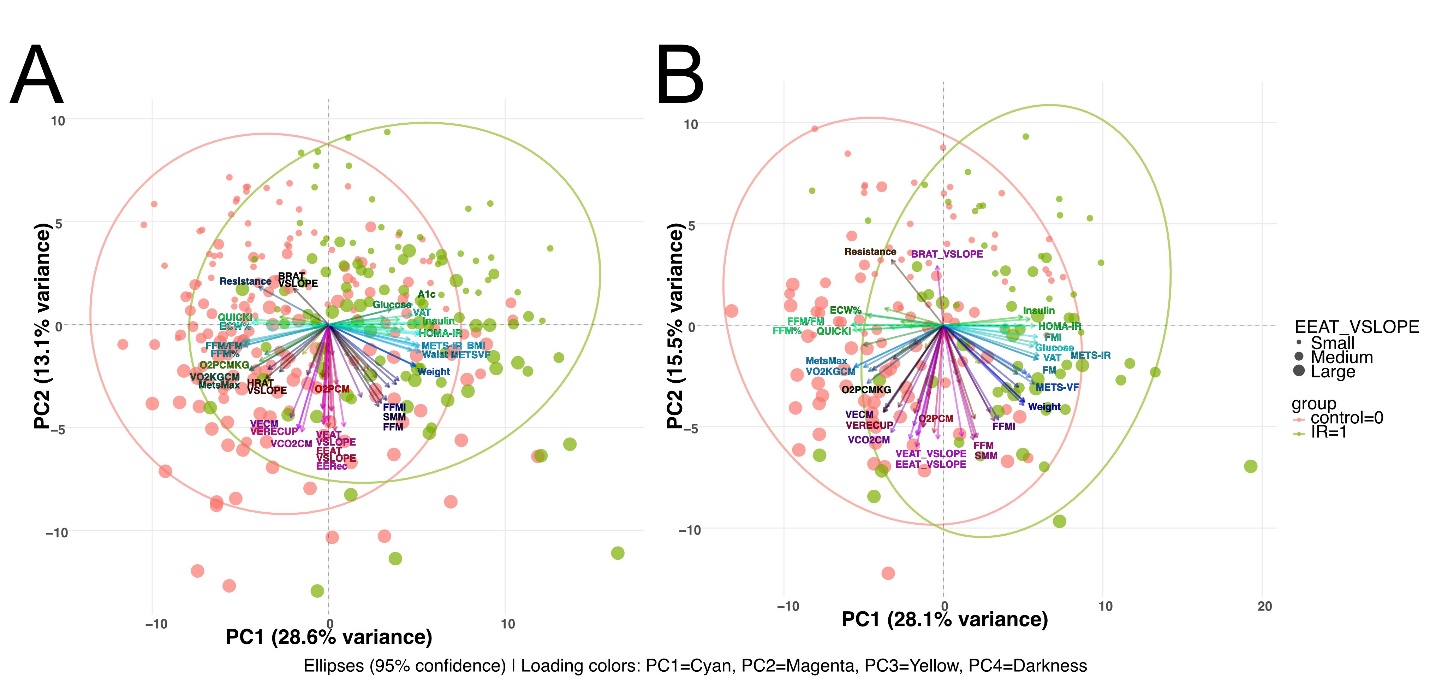


**Suplementary Fig 2.** PCA analysis respect to VO2max. Panel A shows the biplot for females and panel B for males. Principal eigenvectors PC1 vs PC2 includes all variables from each subject. Individuals with insulin resistance were clustered in the green group and those without in the red group with 95% CI ellipses. To show the contribution of each variable towards different components, each vector is colored proportionally. BMI: body max index, FFM: fat free mass, FM: fat mass, GGT: Gamma-glutamyltransferase, gluc: glucose, HbA1c: glycated hemoglobin, HOMA-IR: homeostasis model assessment – insulin resistance, LDL: low density lipoprotein cholesterol, METS-IR: metabolic score insulin resistance index, METS-VF: metabolic score for visceral fat, QUICKI: quantitative insulin sensitivity check index, SMM: skeletal muscle mass, VAT: visceral adipose tissue.


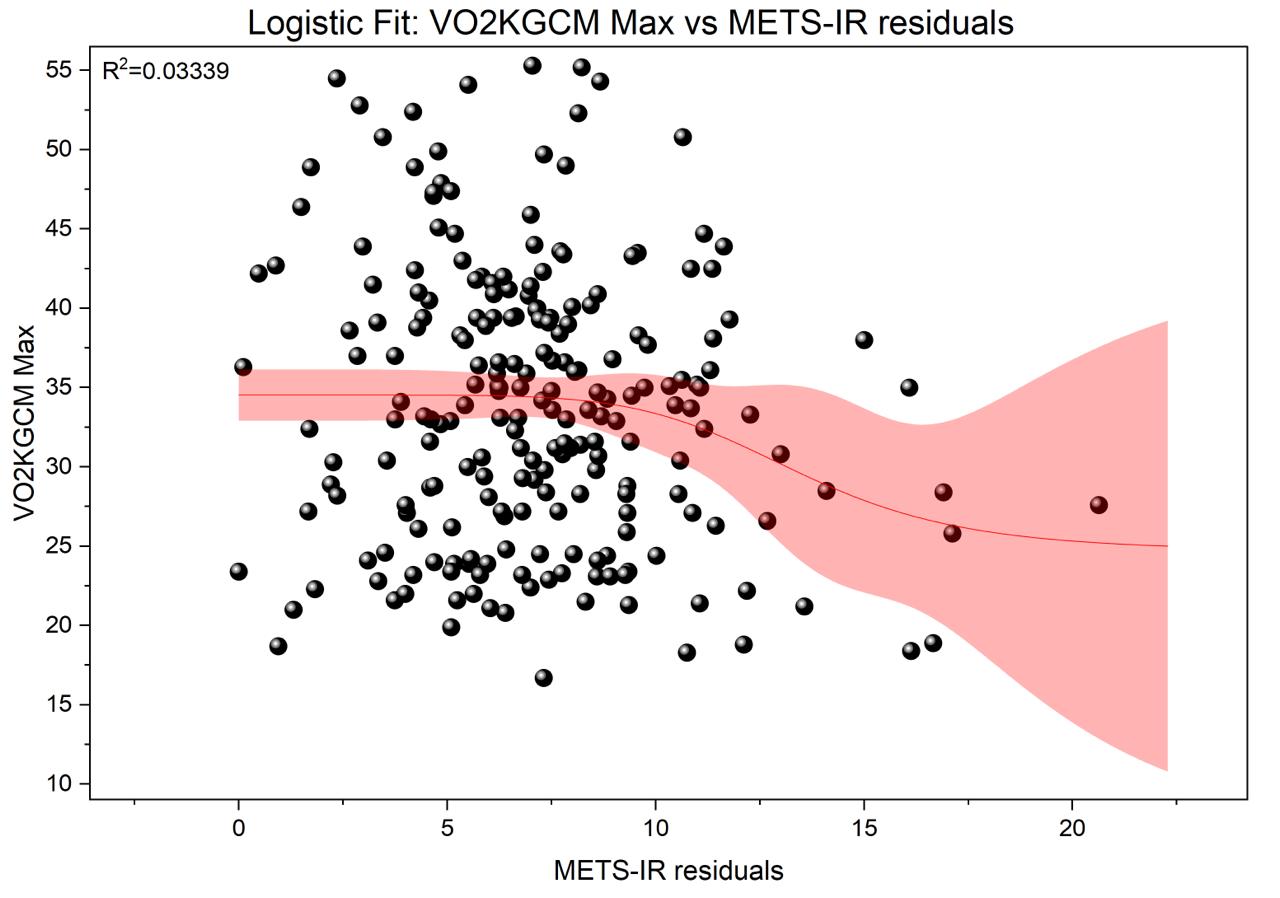


**Supp fig 3**. Logistic FIT: VO2max versus METS-IR residuals. Four-parameter logistic (4PL) fit of VO₂max as a function of BMI-adjusted METS-IR residuals. Residuals were derived from sex-stratified linear regressions of METS-IR on BMI to isolate the adiposity-independent component of insulin resistance. Model selection via F-test and AIC favored the 4PL function over linear regression. The red line represents the best-fit curve (R² = 0.033); shaded area indicates 95% CI. The sigmoidal pattern suggests a threshold effect whereby VO₂max declines at higher levels of residual insulin resistance. BMI residualization was performed given that VO₂max is weight adjusted. The residual variance explained after adjustment, though modest, is consistent with an adiposity-independent association between insulin resistance and cardiorespiratory fitness.
